# Systems, supplies, and staff: a mixed-methods study of health care workers’ experiences and health facility preparedness during a large national cholera outbreak, Kenya 2015

**DOI:** 10.1186/s12889-018-5584-5

**Published:** 2018-06-11

**Authors:** Kathryn G. Curran, Emma Wells, Samuel J. Crowe, Rupa Narra, Jared Oremo, Waqo Boru, Jane Githuku, Mark Obonyo, Kevin M. De Cock, Joel M. Montgomery, Lyndah Makayotto, Daniel Langat, Sara A. Lowther, Ciara O’Reilly, Zeinab Gura, Jackson Kioko

**Affiliations:** 1 US Centers for Disease Control and Prevention, 1600 Clifton Road NE, Mailstop E-04, Atlanta, GA 30329 USA; 2 Safe Water and AIDS Project, Kisumu, Kenya; 3 Ministry of Health, Kenya Field Epidemiology and Laboratory Training Program, Nairobi, Kenya; 4 US Centers for Disease Control and Prevention , Nairobi, Kenya; 5 Ministry of Health, Disease Surveillance and Response Unit, Nairobi, Kenya; 6 Ministry of Health, Department of Preventive and Promotive Health, Nairobi, Kenya

**Keywords:** Kenya, Cholera, Mixed-methods, Decentralization, Devolution, Preparedness, Surveillance, Outbreak response, Global health security

## Abstract

**Background:**

From December 2014 to September 2016, a cholera outbreak in Kenya, the largest since 2010, caused 16,840 reported cases and 256 deaths. The outbreak affected 30 of Kenya’s 47 counties and occurred shortly after the decentralization of many healthcare services to the county level. This mixed-methods study, conducted June–July 2015, assessed cholera preparedness in Homa Bay, Nairobi, and Mombasa counties and explored clinic- and community-based health care workers’ (HCW) experiences during outbreak response.

**Methods:**

Counties were selected based on cumulative cholera burden and geographic characteristics. We conducted 44 health facility cholera preparedness checklists (according to national guidelines) and 8 focus group discussions (FGDs). Frequencies from preparedness checklists were generated. To determine key themes from FGDs, inductive and deductive codes were applied; MAX software for qualitative data analysis (MAXQDA) was used to identify patterns.

**Results:**

Some facilities lacked key materials for treating cholera patients, diagnosing cases, and maintaining infection control. Overall, 82% (36/44) of health facilities had oral rehydration salts, 65% (28/43) had IV fluids, 27% (12/44) had rectal swabs, 11% (5/44) had Cary-Blair transport media, and 86% (38/44) had gloves. A considerable number of facilities lacked disease reporting forms (34%, 14/41) and cholera treatment guidelines (37%, 16/43). In FDGs, HCWs described confusion regarding roles and reporting during the outbreak, which highlighted issues in coordination and management structures within the health system. Similar to checklist findings, FGD participants described supply challenges affecting laboratory preparedness and infection prevention and control. Perceived successes included community engagement, health education, strong collaboration between clinic and community HCWs, and HCWs’ personal passion to help others.

**Conclusions:**

The confusion over roles, reporting, and management found in this evaluation highlights a need to adapt, implement, and communicate health strategies at the county level, in order to inform and train HCWs during health system transformations. International, national, and county stakeholders could strengthen preparedness and response for cholera and other public health emergencies in Kenya, and thereby strengthen global health security, through further investment in the existing Integrated Disease Surveillance and Response structure and national cholera prevention and control plan, and the adoption of county-specific cholera control plans.

## Background

Cholera is an intestinal infection caused by the toxigenic bacterium *Vibrio cholerae* serogroup O1 or O139, and is transmitted through the fecal-oral route [1]. It primarily affects poor populations who lack access to safe drinking water and sanitation [2]. In severe cases, cholera can quickly cause severe dehydration and death; however, with timely treatment, case fatality rates (CFRs) should remain < 1% [2].

On December 26, 2014, the Kenya Ministry of Health (MOH) first detected cases of cholera in Nairobi. As of September 13, 2016, the cholera outbreak in Kenya, the largest since 2010, caused 16,840 reported cases (including 1986 laboratory-confirmed cases) and 256 deaths (national CFR = 1.5%) [3]. The outbreak affected 30 of 47 counties, many of which experienced multiple “waves” or small outbreaks between periods when the outbreak was controlled [4]. According to the World Health Organization (WHO), Kenya accounted for 19% of the 71,176 globally reported cases and 7% of the 937 globally reported deaths (ratio[CFR = 0.5%]) in 2015 [5].

Due to cholera outbreaks in Kenya and throughout sub-Saharan Africa, the WHO, the United Nations International Children’s Emergency Fund (UNICEF), Medecins sans Frontieres (MSF), the Kenya MOH, and other partners have published guidance for detecting, reporting, and responding to cholera outbreaks [6–11]. These guidelines include information on cholera supplies to be stocked in healthcare facilities, case definitions, diagnostic procedures, patient management, and infection prevention and control (IPC) measures. In Kenya, cholera surveillance is part of the Integrated Disease Surveillance and Response (IDSR) strategy, which was adopted in 1998 [12]. According to the International Health Regulations [13] and IDSR guidelines [12], cholera is classified as a disease “with highly epidemic potential” and requires immediate notification for rapid control of an outbreak at its source to ensure global health security. In Kenya, one confirmed cholera case, represents an outbreak and is mandated to be reported within 24 h [12]. For confirmation of a cholera outbreak in a county, Kenya cholera guidelines recommend the collection of a stool sample from the first suspected cholera case-patient for laboratory testing, isolation of *Vibrio cholerae* from a stool culture and determination of O1 serotype [10]. While cholera guidelines have been created, effective implementation is not guaranteed and multiple factors influence the success of response.

This cholera outbreak was the first to occur since constitutional changes that decentralized management of healthcare from a national system, under which the national government provided oversight to provincial and county governments, to a county based system. In 2010, the Kenyan people approved a new constitution that decentralized (or devolved) many aspects of the national executive, legislative, and administrative authority to 47 newly established county governments [14]. This transition was implemented in 2013; county governments became responsible for overseeing most public healthcare facilities, performing local disease surveillance and response activities, and managing water and sanitation services within their counties [15].

This mixed-methods evaluation, including quantitative and qualitative components, aimed to assess cholera preparedness in several counties and explore clinic- and community-based health care workers’ (HCWs) experiences during the 2015 cholera outbreak response.

## Methods

**Fig. 1:**
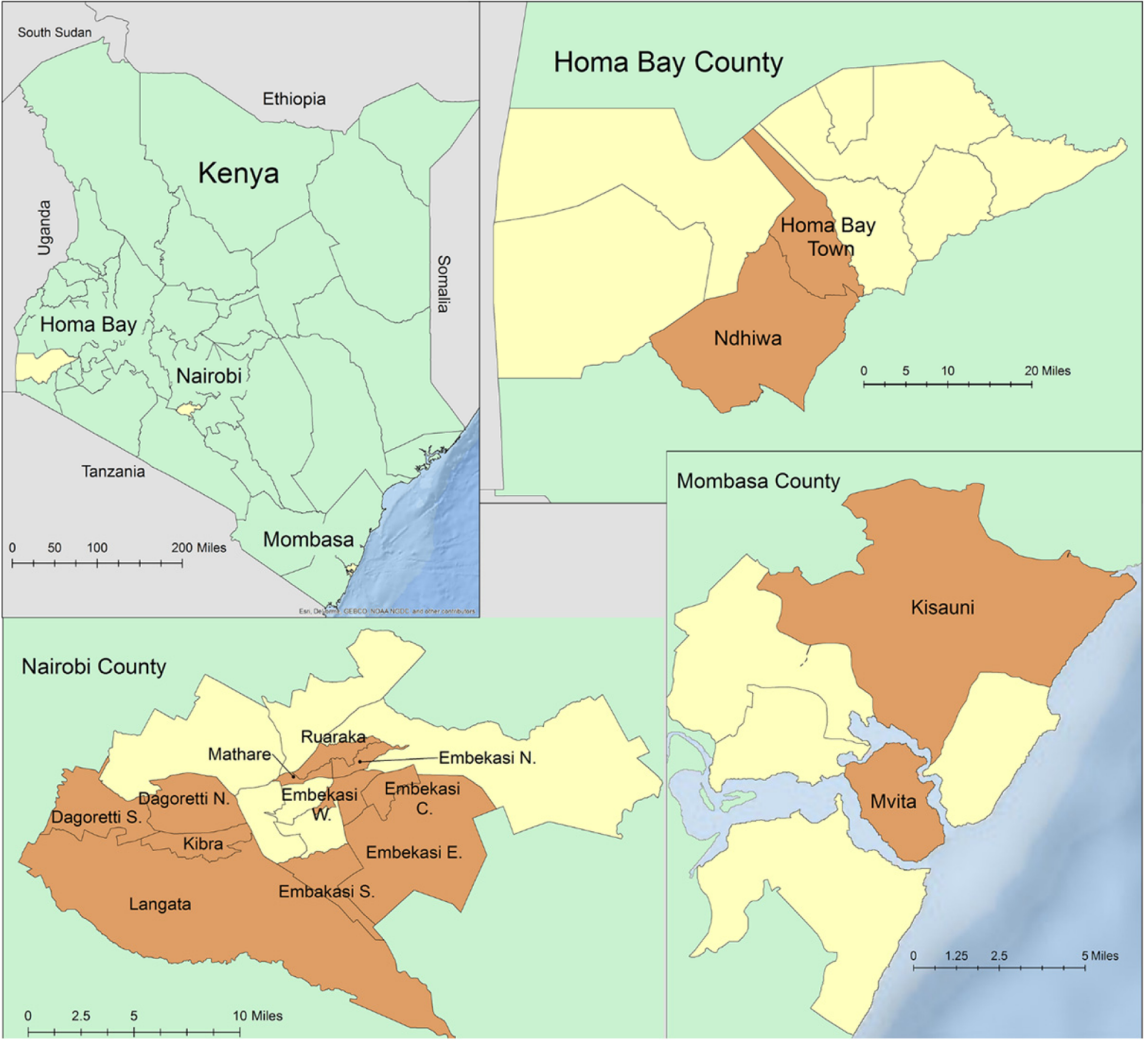
Counties and sub-counties selected for rapid assessment of cholera response, Kenya July 2015. Map of Kenya shows three counties selected for rapid assessment of cholera response in yellow. Homa Bay, Nairobi, and Mombasa County maps show selected sub-counties in orange

**Fig. 2:**
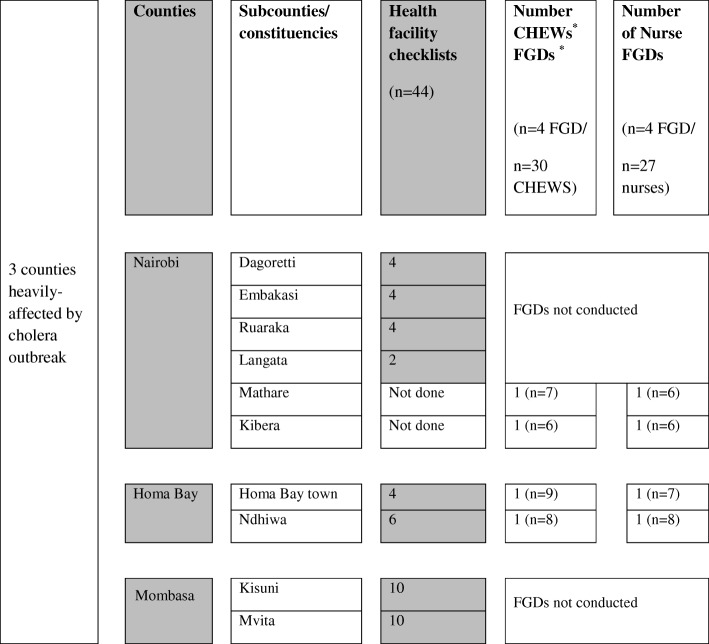
Quantitative and qualitative methods employed during a rapid assessment of cholera outbreak response, Kenya July 2015. Number of health facility cholera preparedness checklists, number of focus group discussions (FGD) completed with community health extension workers (CHEW) and nurses, and number of FGD participants (n) in parenthesis are listed by sub-counties in Nairobi, Homa Bay, and Mombasa Counties

In June–July 2015, a team from the Kenya Ministry of Health’s (MOH) Field Epidemiology and Laboratory Training Program (FELTP) and the Centers for Disease Control and Prevention (CDC) implemented a rapid assessment of cholera preparedness and response in Homa Bay, Mombasa, and Nairobi counties. Counties were selected based on: the cumulative burden of cholera cases and/or cholera deaths during the outbreak; whether an outbreak was ongoing when the study was planned (i.e. cholera cases reported in previous week); and consideration of regional diversity that identified urban and rural areas, and coastal versus non-coastal locations. Two sub-counties in rural Homa Bay (Homa Bay Town, Ndhiwa), two in coastal, urban Mombasa (Kisuani, Mvita), and four in urban, non-coastal Nairobi (Dagoretti, Embakasi, Kasarani, Langata) were selected using the same criterion (Figs. 1 and 2). At the time of the assessment, cholera outbreaks were ongoing in Nairobi and Mombasa, while several weeks had elapsed since the last reported cholera case in Homa Bay.

### Definition of terms

Community Health Extension Workers (CHEWs) are trained facility-based health personnel with a certification in nursing or public health [16]. They train, supervise, and support Community Health Workers (CHWs) and manage community health services records to ensure the quality of community health services. CHWs are community volunteers selected by community members to deliver primary care to households (i.e., common ailments and minor injuries), educate communities on how to prevent illness, and refer cases to the nearest health facility [17]. Nurses are based in healthcare facilities and provide clinical care and education to patients.

### Quantitative data collection

A convenience sample of health facilities was selected from a master health facility list to include various facility types (e.g., government hospital, health center, health dispensary, faith-based facility) in the sub-counties and districts of the three counties (Figs. 1 and 2). Trained data collectors completed cholera preparedness checklists by interviewing health facility staff and directly observing presence of cholera treatment supplies (e.g. Oral Rehydration Solution (ORS), Intravenous (IV) fluids and tubing, doxycycline), laboratory supplies (e.g. rectal swabs, Cary Blair transport media, cholera rapid test kits), IPC supplies (e.g. gloves, chlorine, hand sanitizer, hand washing stations, water source on premise), IDSR guidelines and weekly reporting forms, and national cholera management and treatment guidelines (Table 1). The cholera preparedness checklist was developed from review of international and national guidelines for cholera preparedness [6–10]. According to the Kenya Cholera Control Guidelines, a minimum surveillance package for cholera preparedness includes a rapid response team in place with contingency funds, rehydration fluids (Ringers lactate solution and ORS), antibiotics (doxycycline, erythromycin, or other appropriate antibiotics), and laboratory reagents for cholera confirmation [10]. Data was entered into Epi Info (CDC, Atlanta GA). Frequencies of supply and guideline availability were calculated using STATA (StataCorp, College Station, Texas).

**Table 1:** Cholera preparedness supplies observed in health facilities by county in Kenya, July 2015^a^

	Homa Bay (*n* = 10)		Nairobi (*n* = 14)		Mombasa (*n* = 20)		Total (*n* = 44)	
	n	%	n	%	n	%	n	%
Cholera Treatment Supplies								
Gloves	9	90	14	100	15	75	38	86
ORS	6	60	14	100	16	80	36	82
IV fluids	3	30	14	100	11	58	28	65
IV tubing, adults	8	80	13	93	13	65	34	77
IV tubing, pediatrics	7	70	13	93	9	45	29	66
Doxycycline	8	80	14	100	14	70	36	82
Doxycycline, erythromycin, or other antibiotic	9	90	14	100	16	80	39	89
Laboratory Supplies								
Rectal swabs	2	20	3	21	7	35	12	27
Cary-Blair transport media	0	0	3	21	2	10	5	11
Cholera rapid test kits	0	0	0	0	4	20	4	9
Infection Prevention and Control								
Gloves	9	90	14	100	15	75	38	86
Chlorine for cleaning	5	50	12	86	16	84	33	75
Hand sanitizer	3	30	11	79	9	45	23	52
Outpatient handwashing station	10	100	6	46	17	89	33	79
With soap and water^b^	5	56	3	25	14	82	22	58
Water source on premises	9	90	14	100	15	79	38	88
Guidelines and Reporting Forms								
MOH 505 IDSR Weekly Summary Forms	6	67	7	54	14	74	27	66
IDSR Guidelines	2	20	4	29	1	5	7	16
Cholera Management and Treatment Guidelines	6	60	11	79	10	53	27	63

^a^*ORS* oral rehydration salts, *IV* intravenous, *MOH* Ministry of Health, *IDSR* integrated disease surveillance and response

^b^Proportion calculated based on available observations; denominators may vary due to missing responses

### Qualitative data collection

In July 2015, eight focus group discussions (FGDs) were conducted with a total of 57 HCWs in urban Nairobi county (4 FGDs, 25 participants) and rural Homa Bay county (4 FGDs, 32 participants) (Figs. 1 and 2). A convenience sample of nurses and CHEWs were invited to participate. FGDs of six to nine participants each were conducted with each type of HCW (nurse or CHEW) in two sub-counties in each county. Two Kenyan female ethnographers facilitated the FGDs in English. They followed a standardized guide which probed about HCWs’ experiences during the recent cholera outbreak and with the decentralization of health services.

All FGDs were recorded and transcribed in English. Two qualitative researchers read all transcripts and developed a codebook through an iterative process of transcript review and revision of codes and coded text. Transcripts were coded in qualitative data analysis software MAXQDA (version 11, Berlin, Germany), key concepts were mapped, and analysis memos were written to elucidate key themes [18]. To reach consensus, a team of four investigators met regularly to agree on the codebook, interpretation of codes, and key themes emerging from the FGDs. Also, researchers used “group-to-group validation” to triangulate findings across each focus group and identify patterns by county and HCW type [19, 20]. Qualitative findings were also validated through the team approach and the application of codes by two independent researchers.

### Ethics

This evaluation was reviewed in accordance with human subjects protection policy and was determined to be nonresearch, routine public health activities, by the Human Research Protection Office of the National Center for Emerging and Zoonotic Infectious Diseases (NCEZID) of the Centers for Disease Control and Prevention, Atlanta, GA, USA (determination # 061215CO) and the Kenya Ministry of Health, Disease Surveillance and Response Unit and Department of Preventive and Promotional Health. All participants provided oral informed consent, which was documented by a facilitator before commencing focus group discussions (CDC NCEZID determination # 061215CO).

## Results

### Quantitative surveys: health facility checklists

We surveyed 44 health facilities in three counties: 10 in Homa Bay (among 78), 14 in Nairobi (among 353), and 20 in Mombasa (among 213) (Figs. 1 and 2; Table 1). These facilities included hospitals (12), health centers (14), and dispensaries (18).

Of all health facilities surveyed for cholera treatment supplies, 82% (36/44) had ORS and 65% (28/43) had IV fluids (Table 1). In Homa Bay, Nairobi, and Mombasa respectively, 60% (6/10), 100% (14/14), and 80% (16/20) of health facilities had ORS, while 30% (3/10), 100% (14/14), and 58% (11/19), had intravenous fluids for cholera treatment. Antibiotics were found in 90% (9/10) of health facilities in Homa Bay, 100% (14/14) in Nairobi, and 80% (16/20) in Mombasa.

Few health facilities had sufficient laboratory supplies for cholera confirmation. In Homa Bay, Nairobi, and Mombasa, respectively, 20% (2/10), 21% (3/14), and 35% (7/20) of health facilities had rectal swabs; 0% (0/10), 21% (3/14), and 10% (2/20) had Cary-Blair transport media, which maintains the integrity of clinical specimens to be tested for *V. cholerae* at the laboratory. Rapid diagnostic tests for detection of *V. cholerae* in stool specimens were only available in 20% (4/20) of health facilities in Mombasa County, and were not found in any other county.

The following IPC materials were observed at health facilities visited in Homa Bay, Nairobi, and Mombasa, respectively: gloves (90, 100, 75%), chlorine for cleaning (50, 86, 84%), and hand sanitizer (30, 79, 45%) (Table 1). Among all health facilities, 88% (38/44) had a water source on premises, 79% (33/42) had handwashing stations in the outpatient department, and 58% (22/38) had soap and water observed at the handwashing station.

Overall, 63% (27/43) of health facilities had a copy of cholera management and treatment guidelines. Few health facilities (16%, 7/43) had IDSR guidelines for direction on how to identify and respond to cholera cases, while a larger proportion (66%, 27/41) had the IDSR weekly summary form to track rates of disease. Generally, a greater proportion of high-level health facilities (i.e. hospitals and health centers) had cholera treatment and laboratory supplies, IPC supplies, and cholera management guidelines (Table 2).

**Table 2:** Cholera preparedness supplies observed in health facilities by type of facility in Kenya, July 2015^a^

	Dispensary (*n* = 18)		Health Center (*n* = 14)		Hospital (*n* = 12)		Total (*n* = 44)	
	n	%	n	%	n	%	n	%
Cholera Treatment Supplies								
Gloves	13	72	13	93	12	100	38	86
ORS	12	67	13	93	11	92	36	82
IV fluids	9	50	10	71	9	75	28	65
IV tubing, adults	10	56	13	93	11	92	34	77
IV tubing, pediatrics	7	70	11	79	11	92	29	66
Doxycycline	13	72	13	93	10	83	36	82
Doxycycline, erythromycin, or other antibiotic	14	72	14	100	11	92	39	89
Laboratory Supplies								
Rectal swabs	1	5	1	7	10	83	12	27
Cary-Blair transport media	0	0	1	7	4	33	5	11
Cholera rapid test kits	0	0	0	0	4	33	4	9
Infection Prevention and Control								
Gloves	13	72	13	93	12	100	38	86
Chlorine for cleaning	12	67	11	79	10	83	33	75
Hand sanitizer	6	33	9	64	8	67	23	52
Outpatient handwashing station	17	100	5	36	11	92	33	79
With soap and water^b^	10	59	5	100	7	73	22	67
Water source on premises	13	72	13	93	12	100	38	88
Guidelines and Reporting Forms								
MOH 505 IDSR Weekly Summary Forms	12	67	8	57	7	58	27	66
IDSR Guidelines	1	5	2	14	4	33	7	16
Cholera Management and Treatment Guidelines	9	50	9	64	9	75	27	63

^a^*ORS* oral rehydration salts, *IV* intravenous, *MOH* Ministry of Health, *IDSR* integrated disease surveillance and response

^b^Proportion calculated based on available observations; denominators may vary due to missing responses

### Qualitative study

In response to the questions from the semi-structured focus group guide, participants described their roles during the cholera outbreak response as well as successes and challenges. A main theme that emerged was barriers in the health system, including confusion regarding roles and reporting systems, and overall coordination during the outbreak.

#### Participation in cholera outbreak response

During the focus groups, nurses described their participation in the cholera response as centered on patient care through identification, treatment, and referral of suspected cholera patients. Many established isolation of cholera patients and trained clinic staff on case management and IPC. Nurses led health talks with patients, family members, staff, and CHEWs. Some also procured medical supplies (e.g. antibiotics, ORS, cholera beds) through coordination with district health management teams (DHMT) and facilitated specimen collection and transport to reference laboratories. Some nurses also noted that they provided antibiotic prophylaxis to family members of cholera patients, and worked with CHEWs to conduct contact tracing.

As part of the cholera outbreak response, CHEWs led community education and outreach, including trainings for CHWs, health talks at schools, and distribution of information, education, and communication materials (e.g. cholera posters). Some CHEWs described targeting interventions to cholera hot spots, such as communities where someone had died of cholera or where many cholera treatment center patients lived. Many conducted contact tracing by visiting households and neighbors of cholera patients and distributing antibiotic prophylaxis and water treatment kits. One CHEW described her cholera response work as “connecting the facility with the community” by “ensuring the infected people would reach the hospital.”

#### Health system barriers during cholera response: Roles, reporting, and coordination

In FGDs, HCWs shared experiences during the cholera outbreak response which highlighted health systems barriers, including lack of coordination and communication of response roles and cholera case reporting. Participants from all sub-counties described confusion over the reporting system as well as their designated role and responsibilities during the response. This confusion may have led to delays and inefficiency (e.g. tasks duplicated), which HCWs perceived could have been remedied by improved engagement by their managers.

*“Like you are saying, sometimes you don’t know your roles and responsibilities when it comes to cholera outbreak; it is like it is nobody’s business.” (Nairobi, Nurse)*

Some HCWs noted how widespread confusion regarding roles created inefficiency in the cholera response as some roles were redundant, while others were left unfilled. Despite awareness of these inefficiencies, HCWs felt unable to take action since they were uncomfortable speaking with their managers. According to HCWs, “coordination [was] poor” without clear ranking and roles, and they could not discuss their work with management because “the lower one fears approaching the higher one.”

*“Nowadays with the county system, one thing I am noticing is that maybe people do not know their roles, there is confusion, somebody wants to do [their] job and does not know what it entails…so meanwhile we are waiting and somebody is waiting for the other person, people are just confused. It’s not like the national system and how it used to work. That is why information goes to the county, […] and even your immediate supervisor does not even know about what they ought to know.” (Homa Bay, CHEW)*

As expressed above, some HCWs believed confusion regarding roles and responsibilities during the cholera response was due to the transition from a national health system to a decentralized system managed by county governments. HCW workers expressed the need for clear organization and definitions of roles. One HCW identified having access to “a clear organogram from the national level up to the facility level and even down to the community units” as a way to address the “mix up” and clarify the system and roles.

### Surveillance and reporting systems

FGD participants described frustration regarding the cholera case reporting system. HCWs commented on the lack of a clear reporting chain and concern that reporting the first cholera cases would lead to criticism or job loss. They felt that, as opposed to reporting cholera cases only when asked by the county, “they should be empowered to report” the initial cases they saw in the community. One nurse described trepidation with relaying information about cholera cases from their health facility:

*“You know we are working in the era of devolution [decentralization]. We should not be very quick in giving the information because for you to avoid the implication that might come later… in terms of reporting information, we should be very careful. You are supposed to contact the focal persons who should be able to relay the information. Some people will even distort the information. So we need to have a clear system on how reporting should be done.” (Nairobi, Nurse)*

When involved in reporting, HCWs expressed that they were frustrated by constantly sharing case counts to county representatives without getting updates in return. They noted being asked to report cases to several different people, reflecting a lack of coordination in the reporting system.

*“Sometimes you can have five groups on the same day asking you the same question.” (Homa Bay, CHEW)*

### Coordination and management

Frustrations with the reporting system highlighted some underlying resentment toward management during the cholera response. HCWs perceived that interactions with supervisors were one-way; supervisors requested surveillance data for reports, but did not inquire about clinic needs. One nurse described a perceived disconnect between supervisors and healthcare workers on the ground.

*“Here is a CHEW who is called at night ‘somebody has just collapsed right now, what can we do?’ You are just a CHEW and no one is supporting you. It was very hectic, and there is somebody there who needs a report. You are being told, ‘bring the report, how many are they?’” (Nairobi, CHEW)*

HCWs often felt that their supervisors were unaware of barriers to providing quality health care and managing the outbreak at the community and health facility levels. HCWs described supervisor visits in which they felt blamed for poor outcomes, including cholera deaths that were out of their control. One participant proposed a solution to the perceived disconnect: county health managers “do supportive supervision style and not fault finding.”

#### Cholera outbreak response challenges

Inadequate resources hindered cholera prevention and treatment and challenged HCWs, compounding health system barriers. HCWs described having insufficient resources for prevention, case identification, cholera treatment, IPC, and staffing. One nurse explained frustration and a loss of motivation to work due to inadequate healthcare supplies and uncertainty about salaries. Another nurse expressed a similar sentiment regarding the shortage as “working like your hands are tied.”

“*What I can say is that the county should budget well and act [on] the actions that have been submitted from the sub-counties so that we don’t run out of drugs, we don’t have beddings, we don’t have space. As nurses when we [are] working [in] a facility where you don’t have supplies, then you are working like your hands are tied. When the supervisor comes she/he will ask you what you have done, not knowing that you don’t have any capacity.*” *(Homa Bay, Nurse)*

### Infrastructure for cholera prevention

Both nurses and CHEWs were aware of recommended hygiene practices (e.g. hand washing with soap and water) and that a lack of water and sanitation infrastructure contributed to the spread of cholera. They noted how many communities did not have safe water sources or latrines, with poor communities hit hardest by the cholera outbreak. HCWs expressed frustration with recommending water, sanitation, and hygiene (WASH) interventions to prevent cholera, without having resources to make it feasible for communities. When voicing concern regarding the environmental causes of cholera’s spread to county officials, some HCW felt ignored.

*“We also tried…with the DHMT [District Health Management Team] to go out in the community to find out the source of the cholera and we found very pathetic places…where people do not have toilets and they don’t even have clean drinking water… We are telling people that they should maintain good hygiene, but they don’t have good facilities. They don’t have a toilet, places to wash their hands, and maybe they cannot afford that soap or boiling water, treating the water.”* (Nairobi, Nurse)

### Laboratory preparedness

In several instances, participants noted a need for training in procedures for specimen collection (including rectal swabs), transport, and laboratory testing. Some reported that media or reagents were unavailable for specimen transport and performing tests, or samples would, “not reach KEMRI [Kenya Medical Research Institute reference lab] or they were not well [p] reserved.” This contributed to delays in confirmation of cholera cases and consequentially delayed response.

*“One of the things that brought a lot of cases to come up is that you would find that we get the diarrhea cases and we are told that it has not been confirmed in the lab, so you are not sure of where to report… So 2 weeks down the line you still find that cholera has not been confirmed… We were being told that, ‘who has told you that there is a cholera outbreak?’ And meanwhile people are dying and so you would also shrink only to find that even at the district referral hospital — there is even no reagent to test the outbreak… so by the time the county says that it is cholera, so many people had died.” (Homa Bay, CHEW)*

### Infection prevention and control

Both CHEWS and nurses commented on not being protected and “feared for their lives” while providing care with insufficient IPC supplies (e.g. gloves, boots) at facilities and in community households. Nurses reported difficulties managing cholera patients without infecting other patients, given limited space, staffing, and cholera beds.

*“So in that case there was a lot of cross infection because they were dealing with the cholera patients and also with the antenatal care mothers… with a baby who has just been born.” (Nairobi, Nurse)*.

In another FGD, a CHEW expressed frustration that CHWs are working hard to provide services to the community during the cholera outbreak without having gloves and “cholera kits,” a package of items for cholera care (e.g. gloves, thermometers, ORS, cotton, syringes).

### Staffing

HCWs, especially nurses, identified how “staff was a problem” and worker shortages also contributed to cross-infection due to nurses attending to cholera patients and “again attending to the maternity” or other wards. Beyond IPC, understaffing made nurses feel as if they “were just struggling alone” during the cholera outbreak without support from the county.

*“Staffing is very poor in the sub-county, like, I’m not afraid to say that now I am alone in the facility which I have closed, serving a population of 16,646 and I am one. So you can imagine when there is a cholera outbreak…” (Homa Bay, Nurse)*

Finally, to address the supply and procurement issues, one nurse requested that nurses, especially those with administrative roles, be included in decision making on which supplies, and what quantity, should be purchased for healthcare facilities.

#### Cholera outbreak response successes

HCWs felt successful when they overcame resource limitations and had strong coordination between partners at the clinic and community levels. Specifically, they were proud of collaboration between nurses, CHEWs, and CHWs; well-coordinated patient referrals; and work with community leaders and schools to prevent cholera. HCWs also acknowledged cooperation and support from international partners.

Nurses from all of the sub-counties mentioned the importance of having CHEWS and CHWs on the ground.

*“I want to add that the CHWs, when we were on the ground, they were very helpful, because the minute they noted that there was cholera they went and told the health facility, gave the contact and all those, and they continually did the same.” (Nairobi, Nurse)*

Health education was conducted both in the communities and at healthcare facilities with patients’ families. HCWs felt they effectively reached the community and provided key cholera prevention messages.

*“I will add that the youth education also helped, especially the chief barazas [public community meetings] and also during the school health. Since in the barazas is where we could get the carriers of messages back in the county as you could see mothers come even if they saw just as small diarrhea.” (Nairobi, Nurse)*

In addition, nurses and CHEWs frequently cited contact tracing and administration of antibiotic prophylaxis to family members and neighbors of cholera patients as a success of the cholera response. They regarded contact tracing as important to community outreach, health education, and cholera prevention measures.

*“The contact tracing and health education was the strength of prevention of cholera. During the time we were educating them and giving out the treatment.” (Homa Bay, CHEW)*

These cholera response activities exemplified strong collaboration; CHEWs worked with nurses in the clinic to identify cholera patients and find their contacts. Although the use of prophylactic antibiotic treatment of close contacts of cholera patients is not recommended during cholera response [6, 7], these observed successes reflect how HCWs valued coordination and prevention during the outbreak.

Finally, HCWs were proud of the dedicated efforts and long hours they contributed to the cholera response. One CHEW explained how their passion and willingness to work overtime despite resource limitations contributed to cholera control.

*“If it was not the passion, the spread would have gone up, because without funds you have to have the passion to work on it… the CHEWs worked a lot, we would get calls even at night and that will force you to move to the affected area to save lives” (Homa Bay, CHEW)*

## Discussion

This study evaluated health facility preparedness and HCW experiences during the 2014–2016 cholera outbreak in Kenya; it highlights critical areas for improvement in preparedness and response to cholera and similar public health emergencies. While some health facilities had key cholera preparedness supplies, others facilities lacked essential materials to respond to a cholera outbreak, including supplies to provide life-saving treatment to cholera patients and those necessary to collect and transport specimens to diagnose cholera. HCWs experienced health system barriers, such as confusion over roles, an inefficient reporting system, and poor management. Some HCWs believed unclear reporting procedures and fear of repercussions for reporting cholera contributed to detrimental delays in detection and declaration of the cholera outbreak. Within a broader context, HCWs identified additional challenges to the cholera epidemic: lack of potable water, hygiene, and sanitation infrastructure, low laboratory capacity for case confirmation, insufficient resources for IPC, and understaffing of healthcare facilities. Despite these challenges, HCWs felt they aided in the cholera response through their strong collaboration to provide education and outreach to the communities they served.

The rapid assessment of health facilities and FGDs revealed supply challenges that impacted the availability of cholera treatment, laboratory capacity, and IPC. Prompt laboratory confirmation of cholera among the first suspected cholera cases is critical for declaration of a cholera outbreak and activation of response measures to reduce incidence and mortality. Timely treatment reduces the risk of severe dehydration and death from cholera and should maintain case fatality rates < 1% [2, 7]. Over a year into the outbreak, reported cumulative case fatality rates were > 1% in Nairobi (1.8%), Homa Bay (1.2%), and Mombasa (3.7%), while the duration and repeated “waves” suggest challenges in controlling the outbreak [3]. An assessment of cholera-related deaths during a large outbreak in Haiti identified multiple contributing factors to a reported high CFR (> 6%): patient delays in seeking and reaching health care; health worker shortages; CHWs’ lack of training and experience; and insufficient supplies to treat cholera [21]. Observed and reported supply shortages in Kenya, including laboratory reagents and specimen transport media, likely contributed to delays in cholera outbreak detection in these counties. Without these supplies and a clear reporting system, HCWs described feeling helpless in their ability to confirm cholera cases and respond appropriately. Similarly, HCWs felt they were working “with their hands tied” due to limited IPC and cholera treatment supplies and little influence over the medical supply procurement system. These challenges were identified in previous cholera response evaluations in Kenya: Loharikar et al. (2013) found low rates of supplies for managing cholera in two remote districts with high cholera case fatality rates, and Makayotto et al. (2009) found that fewer than a quarter of health facilities visited (24%, *n* = 31) had laboratory capacity to confirm cholera during the 2009 outbreak [22, 23].

Drawing on experiences with past cholera outbreaks, the Kenya government developed national cholera control guidelines [10] and a national cholera prevention and control plan [24]. After this outbreak was declared, the MOH convened the national cholera outbreak stakeholders meeting on May 21, 2015, where national and county governments signed a joint communique that outlined key tasks to implement [25]. County governments pledged to prepare cholera emergency plans, including “resource mobilization, coordination, surveillance, laboratory confirmation, case management, public education, and WASH;” nevertheless, it is unknown whether this led to formal documentation of county plans for cholera response [25]. This study found varying degrees of utilization and awareness of existing national cholera control guidance and IDSR guidelines across three counties in Kenya. In the qualitative evaluation, HCWs described implementing both appropriate cholera prevention measures (e.g. promotion of handwashing) and potentially inefficient measures (e.g. contact tracing and antibiotic prophylaxis) that diverge from the global and national cholera guidelines [7]. Over half of health facilities had national cholera guidelines and IDSR weekly reporting forms, while fewer than a quarter had the IDSR guidelines. Likewise, confusion with roles and reporting during the outbreak was a central theme of the focus groups with HCWs. A previous evaluation of IDSR reporting practices in a random sample of 348 health facilities in Nairobi County found that “adequate reporting” facilities were more likely to have a designated surveillance focal person, posters showing IDSR functions, and a sufficient supply of reporting forms [26]. These qualities of “model” IDSR reporting facilities, along with adequate resource investment, may be implemented to strengthen IDSR and clarify reporting and response roles. A longitudinal study of IDSR in Uganda concluded that improvements in IDSR enhanced preparedness and capacity to quickly detect and respond to disease outbreaks, as demonstrated by a reduced cholera case fatality rate (from 7% in 2001 to 2% in 2007) [27].

Recent major changes to health system governance in Kenya, specifically devolution or decentralization of health services, may have contributed to these health systems barriers: confusion with roles, reporting, and supply chain. A study in Kilifi, Kenya, among health facility managers identified similar health system challenges, including overwhelming, confusing, and repetitious reporting; lack of clarity in roles and responsibilities; and resource scarcity. These challenges were considered to be ‘caused or exacerbated by devolution as it unfolded’ [28]. Additionally, a literature review of factors influencing CHW performance found examples of shifts to decentralized management related with disorganized management of supplies, supervision, and training, which negatively impact the CHWs’ work [29]. Similarly, in 2013, the medical supply procurement system also changed — counties became responsible for managing funds to procure medical commodities on a ‘pull system’ or ‘demand-driven’ supply system [30]. As a result, counties may spend allotted emergency funds, resulting in insufficient funds to procure supplies in the case of an emergency or unanticipated outbreak. While this evaluation was not designed to evaluate these health system changes and their potential impact on the cholera outbreak, the context of the evolving health system is important to consider and may explain challenges in response coordination and management.

Finally, the qualitative component of this study highlighted critical strengths during the cholera outbreak response: coordination amongst clinic and community-based HCWs on the ground and HCW’s personal dedication to patients and the communities they serve. These intangible “supplies” were considered critical to success. An assessment of public primary healthcare facility managers’ functioning during major health reform in Kenya underscored the “importance of leadership development including the building of critical soft skills such as relationship building” [28]. Also, HCWs proposed achievable solutions to address their frustrations, such as access to an organogram of the county health team and reporting chains, clear instructions of their roles and responsibilities during an outbreak response, supportive supervision, and inclusion of a facility representative in medical supply procurement process.

This evaluation has several limitations to consider. First, it was conducted in a convenience sample of health facilities and HCWs in a few counties and sub-counties, thus findings may not be generalizable to other facilities, sub-counties, and counties. The qualitative component focused on the experience of front line clinic- and community-based HCWs from different areas of the country; the perspective of county health teams, managers, and other stakeholders is absent, as it was beyond the scope of our activities. Cholera preparedness checklists were completed several months into the outbreak, and in some counties after it had been controlled, which would have missed critical shortages that might have occurred before or during the outbreak. Cholera preparedness checklists did not assess facility staff composition, training, and motivation; hygiene organization (e.g., patient isolation rooms); or non-governmental support. These factors should be considered for future cholera assessments.

## Conclusion

International, national, and county stakeholders may strengthen preparedness and response for cholera and other public health emergencies in Kenya through further investment in the existing IDSR structure and national cholera prevention and control plan, as well as the adoption of county-specific cholera control plans. Such investments are vital for ensuring rapid detection and control of outbreaks at their source, and thereby ensuring global health security. As part of their county cholera response strategy, counties may consider their county health budget and procurement plans in order to include adequate emergency outbreak funds. The allotment and process for accessing emergency funds at both the county and national levels should be considered and reviewed with county health teams to ensure awareness among appropriate staff. In focus groups, HCWs expressed frustration that the medicines and supplies they needed for cholera response were not provided; they felt county health teams were not asking for their input in purchasing decisions. HCW morale may be increased and logistics management may be improved through greater engagement of medical staff in the supply procurement process. More broadly, the confusion over roles, reporting, and management found in this evaluation highlights a need during health system transformations to adapt, implement, and communicate health strategies, including public health emergency preparedness, at the county level to inform and train HCWs.

## Abbreviations

CDC: US centers for disease control and prevention
CFR: Case fatality rate
CHEW: Community health extension worker
CHW: Community health worker
CTC: Cholera treatment center
DHMT: District Health Management Teams
FELTP: Field Epidemiology and Laboratory Training Program
FGD: Focus group discussion
HCW: Healthcare worker
IDSR: Integrated disease surveillance and response
IPC: Infection prevention control
MOH: Ministry of health
MSF: Medecins sans Frontieres
ORS: Oral rehydration solution
UNICEF: United Nations International Children’s Emergency Fund
WASH: Water sanitation and hygiene
WHO: World Health Organization
